# Determination of intra-oral surface areas by cone-beam computed tomography analysis and their relation with anthrometric measurements of the head

**DOI:** 10.1007/s00276-020-02530-7

**Published:** 2020-07-11

**Authors:** Z. Assy, C. Klop, H. S. Brand, R. C. Hoogeveen, J. H. Koolstra, F. J. Bikker

**Affiliations:** 1 Department of Oral Biochemistry, Academic Centre for Dentistry Amsterdam, University of Amsterdam and VU University Amsterdam, Room 12 N-37, Gustav Mahlerlaan 3004, 1081 LA Amsterdam, The Netherlands; 2 Department of Oral and Maxillofacial Surgery, Amsterdam UMC Location AMC and Academic Centre for Dentistry Amsterdam (ACTA), University of Amsterdam, Amsterdam, The Netherlands; 3 Department of Oral Radiology, Academic Centre for Dentistry Amsterdam (ACTA), University of Amsterdam and VU University Amsterdam, Amsterdam, The Netherlands; 4 Department of Oral Cell Biology and Functional Anatomy, Academic Centre for Dentistry Amsterdam (ACTA), University of Amsterdam and VU University Amsterdam, Amsterdam, The Netherlands

**Keywords:** Tongue, Palate, Anthropometry, Cone-beam computed tomography

## Abstract

**Purpose:**

Determination of intra-oral surface areas might contribute to our understanding of the physiology of the oral cavity and oral diseases. In previous studies, the intra-oral surface area was determined using a laborious and technically challenging method. Our aim was to develop an easy and non-invasive method to determine the intra-oral surface areas.

**Methods:**

In this study, we used cone-beam computed tomography (CBCT) and digital analysis in 20 human cadavers to determine various intra-oral surface areas, based on digital segmentation. Next, we explored whether there was a relationship between various intra-oral surface areas and anthropometric measurements of the head using Pearson correlation coefficient.

**Results:**

Using CBCT and digital analysis, it was possible to determine various intra-oral surface areas. On average, the total intra-oral surface area was 173 ± 19 cm^2^. Moderate, statistical significant correlations were observed between (1) the length of the head and the palatal surface area, as well as (2) the depth of the head and the surface area of the tongue. These correlations suggest the feasibility of estimating intra-oral surface areas without relying on CBCT imaging.

**Conclusions:**

This study presents a technique for measuring the intra-oral surface areas by CBCT imaging in combination with digital analysis. The results of this study suggest that anthropometric measurements of the head might be used to estimate the surface areas of the palate and tongue.

## Introduction

Knowledge of the integrity and anatomy of the intra-oral surface areas, including the oral mucosa, contributes to a better understanding of the physiology of the mouth and the oral health [23]. In addition, knowledge of the intra-oral anatomy and surface areas is important for therapeutic purposes [23], for example in orthodontic treatment and maxillofacial surgery.

Under healthy conditions, the intra-oral surfaces are covered by a salivary film, which moistens the oral cavity [3, 24]. In this light, the size of the intra-oral surface area has previously been measured to determine the distribution and average thickness of the salivary film covering the teeth and oral mucosa [8, 19, 29]. For this reason, dental impressions were made of all structures (including hard and soft tissue) inside the oral cavity. Then, from these impressions, stone models were produced and covered with aluminum foil. Subsequently, this foil was weighted and surface areas were deduced [8, 19, 29]. This foil technique has been proven to be reproducible [8, 19, 29]. However, the adaptation of the foil onto the models without stretching appeared to be technically challenging; as stretching would possibly lead to thinning of the foil and subsequent underestimation of the surface [8]. Another reported challenge was the difficulty to manually extend the foil completely into all interdental spaces, the labial and buccal vestibular mucosa.

Aiming to provide an alternative method, we performed a study to quantify the intra-oral surface areas using cone-beam computed tomography (CBCT) in combination with digital analysis. This method was inspired by previous studies, in which CBCT was used for soft tissue analysis including determination of the void volume of the oral cavity [5, 11, 18, 28]. Obviously, CBCT involves the use of ionizing radiation, rendering this approach unsuitable for medical care routine. This led to the concept to investigate potential correlations between facial anthropomorphic measurements and intra-oral surface areas. It was, therefore, hypothesized that a relation between anthropomorphic measurements and intra-oral surfaces would potentially enable easy approximation of the intra-oral surface area in a chair-side medical setting, without exposure to radiation. For ethical reasons, we explored this hypothesis on cadavers.

## Materials and methods

### Cadavers

In total, 23 human cadaver heads were provided by the Anatomical-Embryological Laboratory of the University of Amsterdam. All cadavers were testamentary donations of volunteers to this department. The use of the material was in accordance with the Dutch Law (Wet Medisch-wetenschappelijk Onderzoek met Mensen, WMO) and the study was approved by the ethical committee of Academic Centre for Dentistry Amsterdam (ACTA, protocol number 2017011).

Arterial embalming has been used to fixate whole body cadavers [6, 12, 25]. A chemical preservative based on formaldehyde was injected through the femoral artery with slight pressure to prevent deformation of the blood vessels in the head. Afterwards, the head was dissected and preserved in a mixed solution of 16.7% glycerol, 8.3% ethanol, and 0.21% phenol.

The cadaver heads all had a complete oral cavity, with the mandibula, the maxilla, the palate, soft tissues and some teeth present. As metallic restoration materials cause scattering on CBCT images, and hence reduce soft-tissue visualization by loss of contrast resolution and image artifacts [2], all metallic materials were removed prior to CBCT scanning.

Cadavers previously dissected in the intra-oral region or cadavers in which mouth opening was impossible were excluded. In this way, three of the 23 cadaver heads were excluded from this study. In the case of seven cadavers, no information about their sex and age was available. The mean age at death of the remaining 13 cadavers was 83 years (range 70–96 years) with a female–male ratio of 8:5. Prior to analysis, the cadavers were removed from the fixation liquid and air dried in a fume cupboard. Additionally, the oral cavity was dried using cotton rolls (PURE, Akzenta International Sa., Chiasso, Switzerland).

### Analysis of anthropometric measurements

**Fig. 1:**
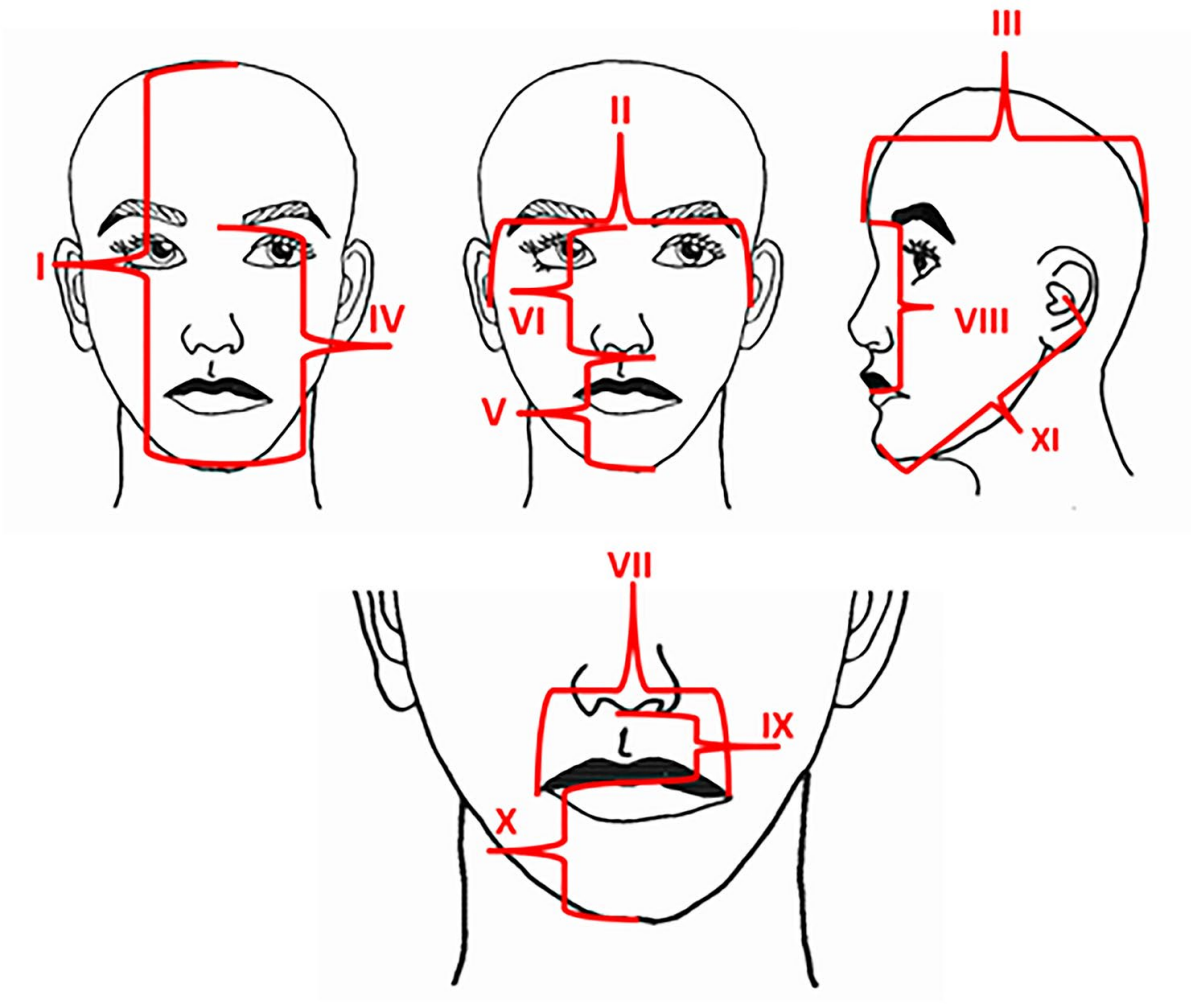
Schematic illustration of human head with all the anthropometric measurements used in this study. Each number indicates a different proportion, see Table 1 for the exact definitions; length of the head (I), width of the head (II), depth of the head (III), face height (IV), lower face height (V), nose height (VI), width of the mouth (VII), upper face height (VIII), upper lip height (IX), mandible height (X) and tragus–gnathion distance (XI)

The distance between anthropometric landmarks was analyzed by two independent measurements using an anatomical sliding caliper (resolution 0.5 mm) which conforms to other studies (Table 1 and Fig. 1) [7, 9, 10, 17, 26].

**Table 1:** Definitions of anthropometric measurements in the present study

Anthropometric measurements	Anthropometric landmark	Illustrated in Fig. 1 as
Length of the head	Vertex–gnathion	I
Width of the head	Straight line distance as measured with sliding caliper between the right external auditory meatus and left external auditory meatus	II
Depth of the head	Straight line distance as measured with a sliding caliper between back of the head and glabella	III
Face height	Glabella–gnathion	IV
Lower face height	Subnasale–gnathion	V
Nose height	Glabella–subnasale	VI
Width of the mouth	Right chelion–left chelion	VII
Upper face height	Glabella–upper lip	VIII
Upper lip height	Subnasale–upper lip	IX
Mandible height	Gnathion–lower lip	X
Mandibular length	Straight line distance as measured with a sliding caliper between the tragus and gnathion	XI
Palatal width	Straight line distance from the upper right first molar (16) to the left first molar (26), if one or both teeth were extracted then the distance from the alveolar ridges of the estimated location of the first molars was used	Not shown

### Removal of metallic restoration materials and preparation of the cadavers for CBCT

After conducting anthropometric measurements, atraumatic extraction of teeth restored with metallic materials was performed to prevent scattering in the CBTC scan. The remaining teeth were all-natural teeth with a total average of 8.7 teeth (SD: 6.2) and a mean number of 5.4 teeth (SD: 3.3) in the lower jaw and 3.3 teeth (SD: 3.8) in the upper jaw. Following extraction, the wet cotton rolls were removed and replaced by six styrofoam bars of approximately 5 × 1 × 1 cm, as styrofoam is undetectable by CBCT and does not absorb fluid in contrast to the cotton rolls. The styrofoam bars were placed in the oral cavity to separate the cheeks and tongue from the oral mucosa and oral gingiva at the following locations: one between the cheek and the lower teeth on the left and right sides, one at both sides between the cheek and the upper teeth and another one was placed between the tongue and teeth at both sides. In some cases where the tongue contacted the palate, additional styrofoam bars were placed between the tongue and the palate. To separate the lips from the frontal teeth, a lip retractor (Henry Schein Dental, Melville, NY, USA) was used [5, 18, 28]. The lip contractor facilitated the insertion of styrofoam bars into the oral cavity but did not influence tissue stretching as the cadavers were preserved in a fixative, which had solidified the tissues.

### CBCT scanning

CBCT scans were acquired using a NewTom 5G CBCT scanner (QR systems, Verona, Italy) at 110 kV, 4 mA, 0.3 mm voxel size and exposure time of 3.6 s. Each cadaver was covered with a plastic bag and was placed inside the scanner as described in the users’ guide. The selected field of view was 12 cm × 8 cm. After selecting patient scan protocol, a regular scan (scanning time 18 s) with a boosted dose was initiated. The scans were saved as Digital Imaging and Communications in Medicine (DICOM) files.

### CBCT analysis

**Fig. 2:**
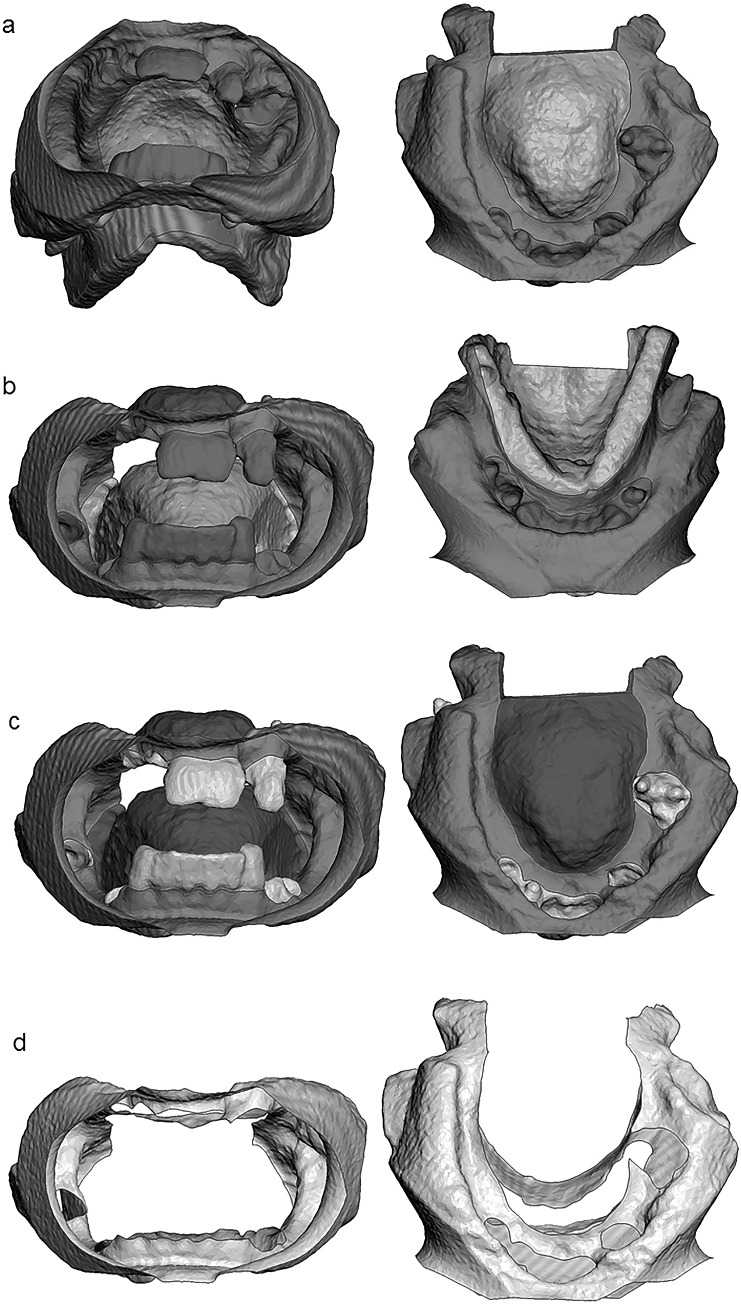
The four different regions segmented in this study from two different views. **a** The palatal surface area is shown in blue color. **b** The tongue surface area is shown in pink color. **c** The hard tissue surface area is shown in green color. **d** The mucosal surface area is shown whereby the palatal, tongue and hard tissue surface areas are made invisible

The DICOM files were reconstructed using Matlab R2019a (Mathworks, Natick, MA, USA). Reconstruction involved segmentation at − 300 Hounsfield units (HU) for soft tissue and 350 HU for bone, filtering with a small smoothing kernel, a morphological closing and conversion to stereolithography (STL) format. Morphological closing is an operation on binary images to remove small gaps while preserving the overall shape and size. In our case, this operation was used to fill small air bubbles in the cadaveric tissue. Subsequently, the STL objects were analyzed in Meshmixer (Autodesk, San Rafael, CA, USA) independently by two researchers (ZA and CK). This analysis involved manual separation of the intra-oral cavity into four regions (Fig. 2): (I) the hard palate, bounded anteriorly and laterally by the maxillary alveolar ridge and posteriorly by the bony pterygoid hamuli. The bony reconstruction was used to determine the positions of the pterygoid hamulus, (II) the tongue, bordered anteriorly and laterally by the mandibular alveolar ridge. Posteriorly, the tongue was limited to the alveolar ridge on the sides and medially to the top view projection of the bony pterygoid hamulus, (III) the hard tissue region was defined as the total of all crowns in situ and dental alveoli of extracted teeth, and (IV) the remaining soft tissue was classified as mucosa, anteriorly limited by the crease of the lip retractor.

After segmentation, the surface areas (in cm^2^) of the four separate regions were determined in Meshmixer.

### Statistical analysis

For statistical analysis, SPSS version 25.0 (IBM Corp SPSS statistics, Armonk, NY, USA) was used. The intraclass correlation coefficient (ICC) was used to determine the degree of agreement between the anthropometric measurements and the oral surface areas. A two-way mixed, absolute agreement, average-measures ICC was calculated for the anthropometric measurements. To measure the agreements between the different researchers for the oral surface areas, a two-way random, absolute agreement, average measures ICC was used [14, 21]. The reliability index is indicative of poor (values less than 0.5), moderate (between 0.5 and 0.75), good (between 0.75 and 0.9) and excellent (greater than 0.90) reliability [20].

The mean of the two anthropometric measurements and the mean of different intra-oral surface areas were used for further analysis. The relationship between anthropometric measurements and the intra-oral surface area was analyzed by a Pearson correlation coefficient. The size of the correlation coefficient was interpreted as negligible (*r* = 0.1–0.2), fair (*r* = 0.3–0.5), moderate (*r* = 0.6–0.7) or very strong (*r* = 0.8–0.9) correlation [1]. An ANOVA one-way test was performed to check for significant differences between females and males. All significance levels (P) were set at 0.05.

## Results

### Intra-oral surface areas

Using CBCT and digital analysis, it was possible to determine the intra-oral surface area. The median of the ICC for the intra-oral surface areas was 0.95 (Table 2). The resulting ICC for the different areas was good or excellent. The mean and standard deviation for the intra-oral surface areas, determined by CBCT and digital analysis, were calculated for the total cadavers, and females and males separately (Table 2). The mean intra-oral surface area of all the included cadavers was 173.3 ± 19.3 cm^2^. ANOVA testing found no significant differences in mean surface areas of the four different regions and the total surface area between females and males.

**Table 2:** The mean and standard deviation of the intra-oral surface area (in cm^2^) for the cadavers, stratified according to gender

Surface area in cm^2^	Total (*N* = 20)	Female (*N* = 8)	Male (*N* = 5)	*P*-value difference female vs male	ICC
Palate	20.0 ± 2.88	20.0 ± 1.78	19.4 ± 4.05	0.748	0.77
Tongue	35.2 ± 5.16	35.0 ± 3.26	34.0 ± 3.67	0.633	0.90
Hard tissue	21.5 ± 11.06	26.4 ± 10.32	15.6 ± 9.76	0.087	0.95
Mucosa	96.6 ± 12.10	94.8 ± 14.55	96.9 ± 12.6	0.792	0.95
Total area	173.3 ± 19.3	176.1 ± 18.6	165.9 ± 18.2	0.353	0.99

*N* indicates the number of cadavers. The *P*-value of the ANOVA one-way test is shown. The ICC indicates the degree of agreements between the different researchers for the oral surface areas. For 7 cadavers the gender was unknown, for this reason they are not included in the ANOVA comparison

### Anthropometric measurements

The ICC for the anthropometric measurements is presented in Table 3. The median of the ICC for the anthropometric measurements was 0.91. The resulting ICC was in the good or excellent range except for two measurements. The length of the head and the mandible height were in the moderate range, indicating less agreement between the first and second measurements.

**Table 3:** The mean and the standard deviation of anthropometric measurements (in cm) for the cadavers, stratified according to gender

Anthropometric measurements in cm (ref Fig. 1)	Total (*N* = 20)	Female (*N* = 8)	Male (*N* = 5)	*P*-value difference female vs male	ICC
Length of head (I)	22.8 ± 0.99 (*N* = 12)	22.3 ± 1.10 (*N* = 5)	23.4 ± 0.95 (*N* = 3)	0.195	0.64
Width of head (II)	15.8 ± 0.89	15.6 ± 0.56	16.6 ± 1.29	0.076	0.96
Depth of head (III)	18.8 ± 0.75 (*N* = 18)	18.5 ± 0.70 (*N* = 8)	19.0 ± 0.48 (*N* = 3)	0.380	0.95
Face height (IV)	14.1 ± 1.04	13.8 ± 0.62	15.1 ± 1.09	0.023	0.96
Lower face height (V)	8.3 ± 0.81	8.2 ± 0.51	8.7 ± 1.16	0.303	0.91
Nose height (VI)	6.1 ± 0.53	5.8 ± 0.44	6.6 ± 0.56	0.018	0.83
Width of mouth (VII)	5.6 ± 0.52	5.7 ± 0.38	5.8 ± 0.51	0.534	0.90
Upper face height (VIII)	8.1 ± 0.65	7.8 ± 0.58	8.7 ± 0.58	0.015	0.91
Upper lip height (IX)	2.2 ± 0.31	2.1 ± 0.25	2.5 ± 0.33	0.021	0.92
Mandible height (X)	4.7 ± 0.61	4.4 ± 0.36	5.2 ± 0.64	0.011	0.66
Mandibular length (XI)	14.7 ± 0.81	14.4 ± 0.47	15.8 ± 0.56	0.001	0.88
Palatal width	4.3 ± 0.31	4.4 ± 0.23	3.9 ± 0.24	0.006	0.96

*N* indicates the number of cadavers. The *P*-value of ANOVA one-way test is shown. The ICC indicates the degree of agreement between the two independent anthropometric measurements. For 7 cadavers the gender was unknown, for this reason they are not included in the ANOVA comparison

Different *N* as in some cases this anthropometric measurements could not be performed

The anthropometric measurements for all the cadavers are also shown in Table 3. The mean and standard deviation for the different anthropometric measurements are presented for all cadavers, female and male cadavers. The results of seven cadavers were not reported separately because their gender was unknown.

Most of the anthropometric measurements showed significant differences between females and males (Table 3). For all measurements, males showed higher values compared to females, except for the palatal width, which was significantly larger in females compared to males.

### Relation between intra-oral surface areas and anthropometric measurements

A significant correlation was found between the surface of the palate and the length of the head, Pearson’s *r*(12) = 0.59, *P* = 0.045. Also, the surface of the tongue and the depth of the head were positively correlated, Pearson’s *r*(18) = 0.50, *P* = 0.036. The Pearson correlation analysis did not reveal significant relations between anthropometric measurements and the total intra-oral surface area (*P* value varying from 0.097 for the palatal width and 0.995 for the upper face height). Also, no significant correlation was found for the surface area of the hard tissue and the mucosa with the anthropometric measurements.

## Discussion

Using CBCT and digital analysis, it was possible to determine the intra-oral surface area. The good and excellent ICCs for the various intra-oral surface areas indicated that this technique is reliable. After the analysis of 20 available cadaver heads, it was found that the average total intra-oral surface was 173 ± 19 cm^2^. In addition, moderate significant correlations between the length of the head and the palatal surface area and between the depth of the head and tongue surface area were observed.

The current study is not the first study to investigate the relationship between extra-oral and intra-oral measurements. Inoue and co-workers found significant correlations between the body profile (especially weight and Body Mass Index) and the salivary gland size [16]. This indicates the possibility to estimate the size of the oral structures by determining extra-oral measurements. In contrast to our study, they found a stronger correlation. A possible reason for this fact could be that they included more subjects (50 young adults compared to 20 cadavers). Another possibility is that some of the cadavers heads included in the current study were incomplete. As a consequence of missing part of the skull (*N* = 8 cadavers), the ICC of the length of the head was moderate. So, the number of included cadavers and the incompleteness of the cadaver heads could have influenced the strength of the correlation between anthropometric measurements and the intra-oral surface area.

The mucosal surface area was found to be 152 ± 16 cm^2^. In comparison, the mucosal surface area found by Naumova and co-workers, who included cadavers of elderly individuals (age 65–75 years), was 197 ± 24 cm^2^ [23]. A possible explanation for this difference might be that, in contrast to our method, Naumova and co-workers used the aluminum foil technique where the outlines of the foils were digitized into AutoCAD [23]. Additionally, they used a profilometer to investigate the dorsal side of the tongue, which measures the tongue surface at high resolution on microscopic level [23]. The dorsal surface of the tongue is covered with lingual papillae which give the tongue an irregular surface texture. As a consequence, the use of this technique may have led to the determination of apparent larger surface areas compared to those found in the present study using CBCT.

Other investigators also used the foil technique to determine the surface area in different regions of the mouth including the teeth. Two studies determining the oral surface areas in infants found that the average total surface area ranged between 118 ± 8 and 143 ± 15 cm^2^, which obviously is smaller than the surface area in the cadavers of the elderly subjects in the current study (173 ± 19 cm^2^) [19, 29]. This age-related increase of the surface area is partly due to the growth of the face and partly to the development of the dentition [15, 27, 29]. Adolescents showed an average intra-oral surface area of 167 ± 13 cm^2^, which is comparable to the findings of the present study [19].

Collins and Dawes also calculated the surface area for twenty living adults using the foil technique [8]. The mean surface area in their study was found to be 215 ± 13 cm^2^, which is larger than the surface area found in the present study, i.e. 173 ± 19 cm^2^. This difference could be attributed to the contribution of the teeth surface area to the total area. Collins and Dawes included subjects having an average of 28 teeth; whereas, the cadavers in this study had an average of 8.7 teeth. For this reason, the surface area of the teeth in the study of Collins and Dawes (45 ± 5 cm^2^) is approximately twice the surface area of all the hard tissue measured in the present study (22 ± 11 cm^2^).

In accordance with the present study, Collins and Dawes found comparable surface areas for the mucosa and the palate [8]. The mean surface areas of the total mucosa and palate their study were 96 and 20 cm^2^, respectively, which is comparable to the present study. However, the surface area of the tongue differed from our study as Collins and Dawes found a surface area of 52 cm^2^ compared to 35 cm^2^ in the present study. Possibly, these differences may be caused by the incomplete measurement of the posterior tongue surface and variation in mouth opening of the cadavers. In some cases, the posterior tongue was not completely separated from the palate with the concomitant risk of missing data on the CBCT scan. Due to limited access to the oral cavity, it was not possible to verify whether the posterior part of the tongue was completely separated from the palate. Additionally, in the current study the length of the tongue was determined by a line on the dorsum of the tongue, corresponding to the bony pterygoid hamuli. However, the cadavers varied in mouth opening, which seemed to introduce variation in the length of the tongue.

Consistent with the present study, Collins and Dawes found no significant gender differences in the surface areas for any of the intra-oral regions [8]. The current study revealed a significant difference in some face proportions between females and males. This finding is broadly supported by the work of other studies describing the effects of gender on anthropometric orofacial measures, mentioning larger measures for males when compared to females [13, 22, 30].

This study has also some potential limitations. It has to be taken into account that the upper part of the palate was imaged incompletely in some cadavers due to a limited field of view. The missing data were reconstructed in Meshmixer by flat filling the defect, for this purpose the “Inspector” analysis tool of Meshmixer was used. Given this fact, the surface area of the palate can be considered a calculated approximation for some cadavers. However, on average, the palatal surface area found in the present study (20.0 cm^2^) was in accordance with two other studies (ranging between 18.0 and 20.1 cm^2^) [8, 19].

Besides, it has to be noted that soft tissues of living persons are more flexible compared to those of embalmed cadavers. In line, several articles mention that embalming procedures following Thiel’s method (main component boric acid) or Imperial College London soft-preservation (main component 80% phenol) give better flexibility and tissue quality than other methods [4, 6, 12, 25]. The embalming technique in the current study might have led to the solidification of the soft tissues.

## Conclusion

The current study presents a reproducible technique for the determination of intra-oral surface areas using CBCT and digital analysis. In addition, this study indicates that moderate, but statistical significant, correlations exist between (1) the length of the head and the palatal surface area, as well as (2) the depth of the head and the surface area of the tongue. Based on these findings, we postulate that it would be possible to estimate individual intra-oral surface areas in situ by measuring facial features.
